# Pod Dehiscence in Hairy Vetch (*Vicia villosa* Roth)

**DOI:** 10.3389/fpls.2020.00082

**Published:** 2020-03-03

**Authors:** Lisa Kissing Kucek, Heathcliffe Riday, Bryce P. Rufener, Allen N. Burke, Sarah Seehaver Eagen, Nancy Ehlke, Sarah Krogman, Steven B. Mirsky, Chris Reberg-Horton, Matthew R. Ryan, Sandra Wayman, Nick P. Wiering

**Affiliations:** ^1^ Dairy Forage Research Center, USDA-ARS, Madison, WI, United States; ^2^ Sustainable Agricultural Systems Laboratory, Beltsville Agricultural Research Center, USDA-ARS, Beltsville, MD, United States; ^3^ Crop and Soil Science, North Carolina State University, Raleigh, NC, United States; ^4^ Department of Agronomy and Plant Genetics, University of Minnesota, St. Paul, MN, United States; ^5^ Noble Research Institute, Ardmore, OK, United States; ^6^ School of Integrated Plant Science, Cornell University, Ithaca, NY, United States

**Keywords:** pod dehiscence, germplasm characterization, vetch, domestication syndrome, phenotyping, legumes (Fabaceae)

## Abstract

Hairy vetch, *Vicia villosa* (Roth), is a cover crop that does not exhibit a typical domestication syndrome. Pod dehiscence reduces seed yield and creates weed problems for subsequent crops. Breeding efforts aim to reduce pod dehiscence in hairy vetch. To characterize pod dehiscence in the species, we quantified visual dehiscence and force required to cause dehiscence among 606 genotypes grown among seven environments of the United States. To identify potential secondary selection traits, we correlated pod dehiscence with various morphological pod characteristics and field measurements. Genotypes of hairy vetch exhibited wide variation in pod dehiscence, from completely indehiscent to completely dehiscent ratings. Mean force to dehiscence also varied widely, from 0.279 to 8.97 N among genotypes. No morphological traits were consistently correlated with pod dehiscence among environments where plants were grown. Results indicated that visual ratings of dehiscence would efficiently screen against genotypes with high pod dehiscence early in the breeding process. Force to dehiscence may be necessary to identify the indehiscent genotypes during advanced stages of selection.

## Introduction

Hairy vetch, *Vicia villosa* (Roth), is an outcrossing diploid legume (2n = 14; Chooi, 1971; Yeater et al., 2004). Commonly used as a green manure cover crop (SARE et al., 2015; CTIC et al., 2016; USDA-NASS, 2019), the species excels in winter hardiness (Brandsæter et al., 2002) and nitrogen supply to subsequent cash crops (Parr et al., 2000). With prevalent pod dehiscence (PD) and seed dormancy, hairy vetch does not exhibit a typical domestication syndrome (Meyer and Purugganan, 2013; Abbo et al., 2014). PD raises seed costs and causes weediness in fields, reducing utilization of this cover crop. Breeding efforts are underway to reduce or eliminate PD in hairy vetch. The goal of such efforts is to increase cover crop use and improve soil and water conservation.

Few published studies have evaluated PD in the genus *Vicia*. Renzi et al. (2017) documented 15% to 46% PD in one Argentinian landrace evaluated at one location over two years, but no studies have evaluated PD of hairy vetch among diverse germplasm or growing conditions. In common vetch (*Vicia sativa* L.), PD varied widely (3% to 96%) among diverse lines (Abd El-Moneim, 1993; Dong et al., 2017). Common vetch lines differing in PD exhibited 22 differentially expressed unigenes (Dong et al., 2017).

In other members of the Fabeae tribe, domestication has successfully eliminated PD (e.g. *Pisum* sp.) or reduced PD to very low levels relative to wild types (e.g. *Lens* sp.; Abbo et al., 2014; see review by Ogutcen et al., 2018). PD was controlled by one to three dominant loci in lentil (*Lens* sp.; Ladizinsky, 1979; Ladizinsky, 1985; Fratini et al., 2007) and one to two dominant loci in pea (*Pisum* sp.; Blixt, 1972; Weeden et al., 2002; Weeden, 2007). PD has been more extensively studied in the Phaseoleae tribe of Fabaceae. In soybean (*Glycine max* L. Merr.), the transcription factor *SHATI-5* and the gene *Pod dehiscence 1* (*Pdh1*) meditate and control PD. In common bean (*Phaseolus vulgaris* L.), various QTL have been identified among bean races (Parker et al., 2019), the most documented being the *Stringless* (*St*) gene in snap beans (Koinange et al., 1996).

PD is influenced by environmental conditions, length of pod drying, and handling methods postharvest (see reviews by Ogutcen et al., 2018 and Zhang et al., 2018). With varying maturity timings, diverse genotypes can be exposed to differing weather conditions during pod development. Consequently, genotype by environment interactions (Tukamuhabwa et al., 2002; Helms, 2011) can cloud genetic effects. More controlled measurements of PD, such as oven drying of pods to standardize moisture and/or applying force to a pod to induce dehiscence were more associated with genetic effects than measuring PD under field conditions (Weeden, 2007; Dong et al., 2016; Murgia et al., 2017; Parker et al., 2019). Such methods, particularly measuring the force needed to induce dehiscence, demand substantial phenotyping time and specialty equipment. Identification of traits that are easier to measure and highly correlated with PD could improve breeding efficiency (Falconer, 1981). Such secondary selection traits could accelerate improvement of hairy vetch.

The objectives of this research were to characterize PD among diverse genotypes of hairy vetch, and to identify the most efficient phenotyping methods for PD. First, we assessed PD among 606 genotypes of hairy vetch in seven environments of the United States. Second, we correlated potential secondary selection traits with measures of PD including visual PD, force to induce dehiscence, and pod spiraling.

## Materials and Methods

### Data Collection

The germplasm for our genetic analysis of PD originated from an existing hairy vetch breeding program. For further details on source germplasm, nursery design, field data collection, and management see Kissing Kucek et al. (2019). In the late summer and early fall of 2017, seven nursery environments (Clayton, NC; Goldsboro, NC; Beltsville, MD; Ithaca, NY; Varna, NY; Prairie Du Sac, WI; and St. Paul, MN) planted half-sib seed from 92 maternal plants selected in the summer of 2017, in addition to 12 check lines (**Table S1**). Fifteen lines were replicated at all environments, including three maternal lines from the 2017 nurseries, four commercially available cultivars, one breeding line, and seven PI accessions. The remaining 77 lines at each site were half-sibling progeny of maternal plants selected from the breeding program in 2017. The seven environments planted a total of 14,304 genotypes of hairy vetch.

Individual plants were screened for visual vigor on a scale from zero to nine in the fall and spring (see Kissing Kucek et al., 2019 for details). When 50% of lines had begun to flower in each nursery, collaborators recorded maturity of each individual plant in the field using the Kalu and Fick (1981). In the spring of 2018, each site selected the top 53 to 122 hairy vetch plants based on winter survival, flowering time, and fall and spring vigor. The selected individuals cross pollinated at each site. Sites recorded the dry weight of selected plants at pod maturity, except Clayton, NC and Prairie du Sac, WI. All sites measured the seed yield of individual plants.

Collaborators harvested a subset of pods from a total of 606 selected plants for PD evaluation. These 606 plants are subsequently referred to as “genotypes.” Collaborators at each site were instructed to collect a subset of around 50 mature pods from the selected maternal plant. If pods appeared to contain few seeds per pod, more pods were collected per plant, to obtain enough seed for a separate analysis on germination. **Figure S1** shows the distribution of pods collected per maternal line per site.

Pods were stored in paper bags under low humidity (< 35% R.H.) conditions at 20°C for five months. A calibration experiment determined that drying pods for least 24 h at 30°C was necessary to stabilize the PD trait and reach critical pod moisture in *Vicia villosa* (see supplementary material and **Figure S2**). Consequently, pods were dried above 30°C for three days to stabilize moisture and PD prior to analysis. Maternal lines were evaluated for metrics of PD and morphology (**Table 1**) in a completely randomized order.

**Table 1:** Metrics of pod dehiscence evaluation, including photos of pods exemplifying the maximum and minimum of rating scales.

Type	Metric	Rating Scale		
Dehiscence Metric	Visual Dehiscence	0 = fully intact pod (no openings along sutures), 1 = one suture was partially opened (one side of pod), 2 = two sutures were partially opened (both sides of pod), 3 = pod had opened in full or partially	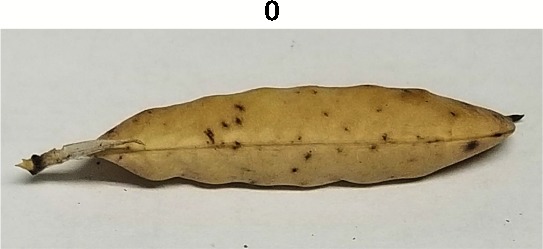	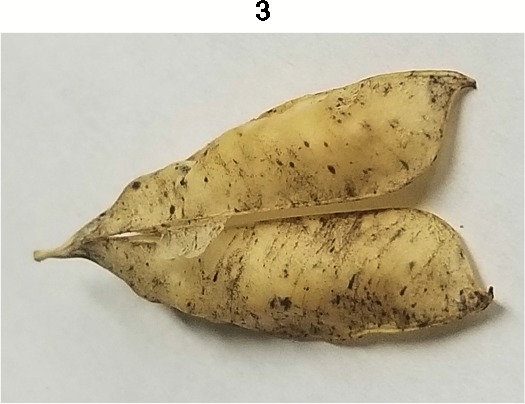
	Force to Dehiscence	peak force (N) applied to create a break in the pod suture		
	Spiraling	0 = no spiraling of pod, 1 = some spiraling of pod, 2 = strong spiraling of pod	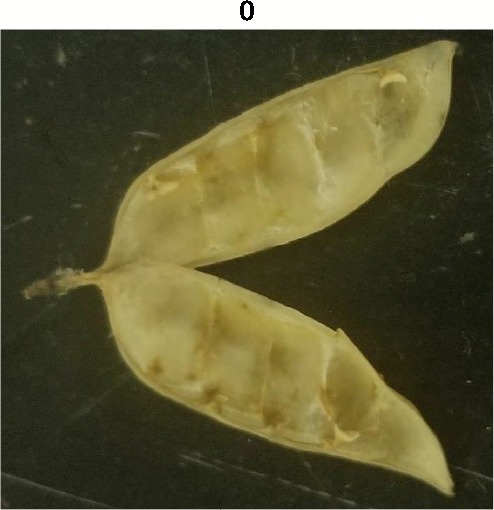	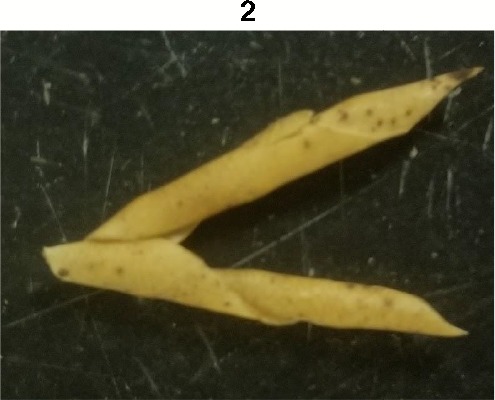
Morphology Metric	Corrugation	0 = smooth on the surface of the pod, 1 = intermediate, 2 = ridged on the surface of the pod - seeds evident	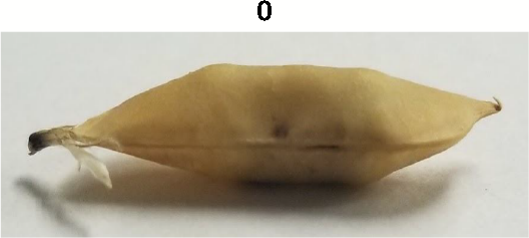	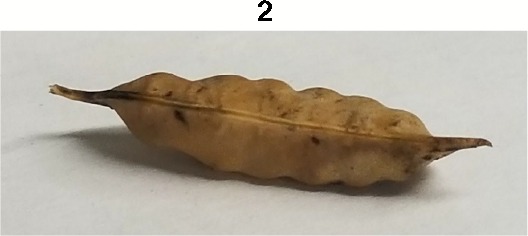
	Fracture	0 = nonlinear fracturing of pod wall when broken, 1 = linear fracturing of pod wall when broken	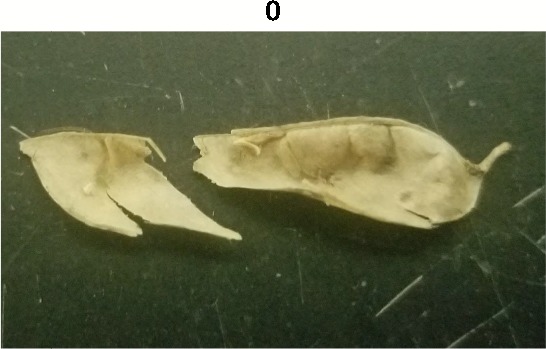	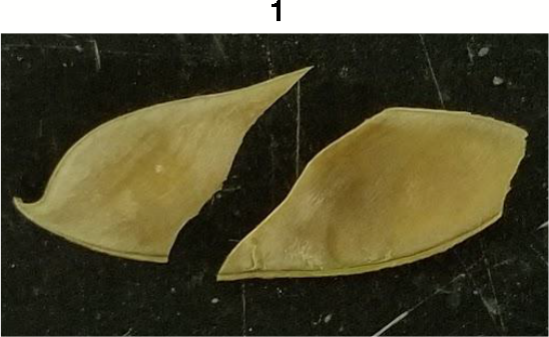
	Flexibility	1 = flexible, 0 = sturdy		
	Pith Tissue	0 = absence of pith tissue inside of pod, 1 = presence of pith tissue inside of pod	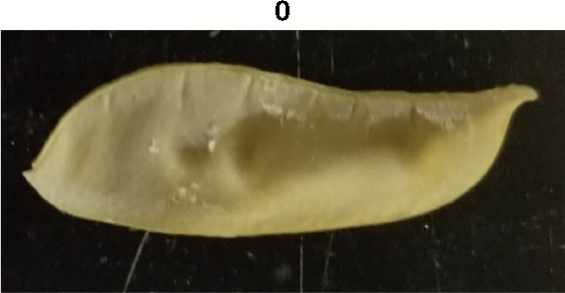	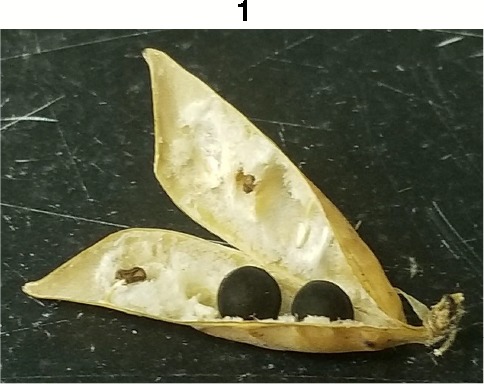

For each pod per maternal line, visual dehiscence was scored on a 0–3 scale, with zero indicating a fully intact pod (no openings along sutures), one indicating one suture was partially opened (one side of pod), two indicating two sutures were partially opened (both sides of pod), and three indicating that the pod had fully opened.

Pods with a zero rating (unopened) were further tested with an MTS Insight ^®^ 1kN (MTS Systems Corporation) machine for measuring cracking force on unopened pods. The pod was placed horizontally on a bottom compression platen, similar to the methods of Dong et al. (2016), and crushed with a top platen until the machine detected peak force required for suture rupture. The evaluation used a 100 N load cell and TestWorks ^®^ [v.4.11B] (MTS Systems Corporation) set to 95% break sensitivity, 0.1 N break threshold, and 25 Hz data acquisition rate. Maternal lines were only evaluated if they had at least three unopened pods available for testing.

After pods were hand-threshed between two ribbed rubber surfaces, the degree of spiraling was rated by the tightness of the curls formed in the carpels. A zero was given to pods with no curling of the carpels, one for intermediate curling, and two for very tight curling of the carpels.

Pods from each maternal line were further rated for morphological features. Traits included corrugation, fracture of the wall, flexibility, and pith tissue (**Table 1**). Prior to threshing, corrugation was visually rated by the compression of the pod wall on the seeds inside the pod. Pods were rated zero if no signs of indents formed by the seeds in the pod wall, one if some indenting was present, and two if the pod wall had significant indentation formed by the seeds. After threshing, pod walls were rated for fracture structure by hand bending the carpel until a break formed. The break would be categorized as zero for pods that formed a jagged fracture, and one if the break followed a straight path. Flexibility was tested in a similar method to fracture. Pods received a zero if they displayed resistance when bending the carpel, and one if the carpel bent with little resistance. After threshing, pods were observed for the presence or absence of a spongy white pith tissue inside of the pod. Pods were given values of zero if there was little to no pith tissue, and one if pith tissue was prevalent.

To verify that differences in pod moisture did not impact PD, a subset of lines diverging for visual PD and force to PD were analyzed for percent moisture. Three lines each from high and low PD categories from each site were included, for a total of 42 lines based on visual dehiscence scores and 40 lines based on force to PD. Some lines overlapped, leaving a total of 76 lines analyzed. Threshed pods, without seeds, were weighed, then dried at 105°C for 24 h and reweighed.

### Statistical Analysis

**Figure 1:**
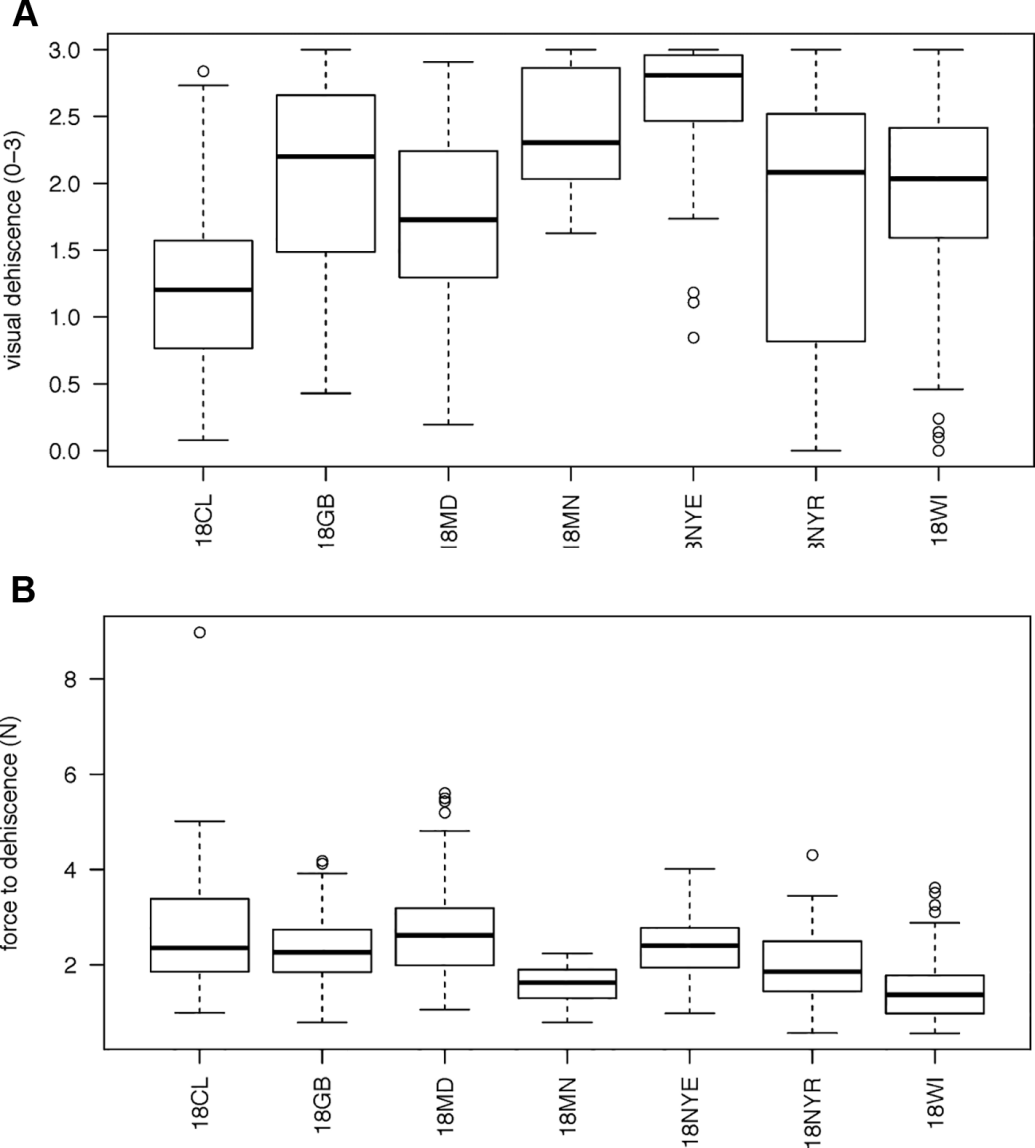
Boxplots of variation in vetch genotypes for **(A)** mean visual dehiscence **(B)** force necessary to cause dehiscence. Environments are listed as 18CL (Clayton, NC), 18GB (Goldsboro, NC), 18MD (Beltsville, MD), 18MN (St. Paul, MN), 18NYE (Ithaca, NY), 18NYR (Varna, NY), 18WI (Prairie Du Sac, WI). There is a strong outlier for force to dehiscence at 18CL. Visual dehiscence was scored on a 0–3 scale, with “0” indicating a pod fully intact pod (no openings along sutures), “1” indicating one suture was partially opened (one side of pod), “2” indicating two sutures were partially opened (both sides of pod), and “3” indicating that the pod had opened fully or partially.

**Figure 2:**
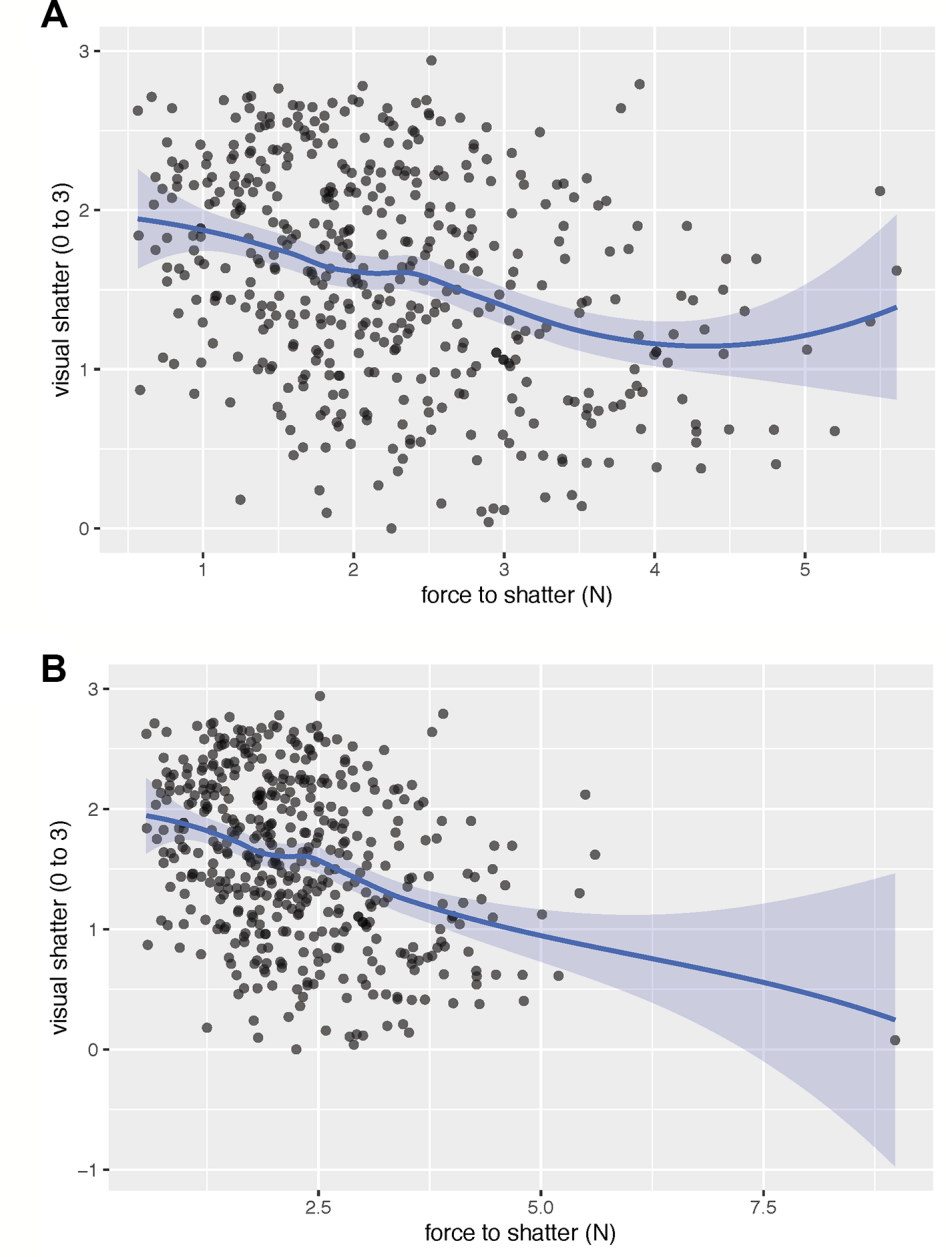
The fitted spline shows the relationship between visual rating of dehiscence and the force required to cause dehiscence, excluding **(A)** and including **(B)** an extreme outlier for force to dehiscence. When excluding the outlier, there was a moderate linear relationship (r = −0.33) between visual dehiscence and force to dehiscence. The linear relationship broke down at low levels of visual dehiscence and high force required to cause pod dehiscence. Both traits may be necessary to identify lines most resistant to dehiscence. Visual dehiscence was scored on a 0–3 scale, with “0” indicating a pod fully intact pod (no openings along sutures), “1” indicating one suture was partially opened (one side of pod), “2” indicating two sutures were partially opened (both sides of pod), and “3” indicating that the pod had opened fully or partially.

Analyses investigated the relationships between all metrics of PD (visual dehiscence, force to dehiscence, and pod spiraling), pod morphology (corrugation, fracture, flexibility, pith tissue), field measurements (fall vigor, spring vigor, maturity timing, plant weight, and seed yield), and pod moisture. Mean values per line were used for visual dehiscence and force to dehiscence. Lines with fewer than four pods observed were removed from analyses. One extreme outlier for force to dehiscence (see **Figures 1B** and **2B**) was also removed prior to analysis.

Line mean visual dehiscence, force to dehiscence, maturity timing, and pod moisture were continuous variables. Corrugation and pod spiraling were ordinal variables artificially created to represent underlying continuous normal distributions, while fracture, flexibility, and pith tissue were dichotomous variables created to represent underlying continuous normal distributions. Maximum likelihood estimates and standard errors of correlations were computed using Pearson's product-moment correlation coefficient (r) for continuous vs. continuous metrics, polyserial maximum likelihood estimates (ρ) for continuous vs. dichotomous or ordinal metrics, and polychoric maximum likelihood estimates (ρ) for dichotomous or ordinal vs. dichotomous or ordinal metrics using the ‘polycor' package (Fox, 2016) in R [version 3.6.0] (Olsson, 1979; Olsson and Drasgow, 1982; R Core Team, 2019). To visualize potential nonlinear relationships between visual dehiscence and force to PD, smoothing splines were generated using the ‘loess' function in R [version 3.6.0] (R Core Team, 2019) and plotted using ‘ggplot2' (version 3.0.0) (Wickham, 2016).

Relationships were tested for difference from zero using Pearson's product-moment correlation for continuous vs. continuous metrics, Kruskal-Wallis one-way analysis of variance for continuous vs. dichotomous or ordinal metrics with nonnormal distribution, one-way analysis of variance (ANOVA) for continuous vs. dichotomous or ordinal metrics with normal distribution, and chi-squared test of independence for dichotomous or ordinal vs. dichotomous or ordinal metrics.

For each combination of metrics, we tested whether correlations varied among environments. Using the ‘psych' package [v.1.8.12] (Revelle, 2019) in R [version 3.6.0] (R Core Team, 2019), independent correlations among environments for each trait combination were z-transformed. The difference between the z-transformed correlations was divided by the standard error of the difference of the z scores. All analyses were conducted in R [version 3.6.0] (R Core Team, 2019), using a significance threshold of P < 0.05.

To further understand the contribution of pod morphology and field maturity to PD, visual dehiscence and force to dehiscence were regressed onto pod morphology and maturity timing using the model below. All effects were treated as random to determine variance contribution of each effect. Variances were estimated using the ‘lme4' package [version1.1-10] (Bates et al., 2015) in R [version 3.6.0] (R Core Team, 2019).

$$Y_{ijklmn}\;=\mu \;+\;\alpha_{i}+\;\beta_{j}+\;\gamma_{k}+\;\delta_{l}+\;\zeta_{m}+\;\eta_{n}+\;\epsilon_{ijk}$$

Y_ijklmn_: visual or force to dehiscence of a pod from environment i, corrugation j, fracture k, flexibility l, pith tissue m, and flowering maturity n

μ: grand mean of visual dehiscence or force to dehiscence

α_i_: random effect of environment i

β_j_: random effect of corrugation j

γ_k_: random covariate of fracture k

δ_l_: random covariate of flexibity l

ζ_m_: random covariate of pith tissue m

η_n_: random covariate of flowering maturity n

ϵ_ijklmn_: error term

## Results

606 genotypes varied from completely indehiscent to completely dehiscent (**Figure 1A**). Twenty genotypes, sourced from five environments, created pods that all opened (mean visual score = 3). In two genotypes, sourced at Prairie du Sac, WI and Varna, NY, all pods were fully closed (mean visual score = 0).

A total of 458 genotypes had at least three unopened pods available for force testing. Mean force to dehiscence varied from 0.279 to 8.97 N among maternal lines (**Figure 1B**). One outlier genotype was evident from the Clayton, NC site. Force to dehiscence for this line was four times the grand mean. Nine of ten pods evaluated for this line were in the 99^th^ percentile of force to dehiscence measured in the study, requiring between 6.2 and 12 N to break the sutures. This line also exhibited the lowest visual dehiscence from its growing location, indicating its promise as a genetic oddity for PD.

Pod moisture varied from 6.7% to 9.3% in a subset of 76 divergent lines for PD. Pod moisture was not significantly related to visual dehiscence, force to dehiscence, flowering maturity, nor pod morphology traits (**Table S2**). Pod moisture showed a weak inverse correlation with spiraling (ρ = -0.25, p = 0.02048). As a result of low influence of moisture in the extreme phenotype subset, pod moisture was not included in further analyses, nor evaluated on the remaining 535 lines.

Corrugation of the pods and presence of pith tissue on the interior of the pod were only common at the three southern nurseries, and rare in the northern nurseries. Northern nurseries were subsequently removed from corrugation correlation calculations. Pod walls at one site (Ithaca, NY) nearly all fractured linearly, and therefore, pods from Ithaca, NY were removed from correlation calculations for fracture. Flowering maturity did not strongly differ at Beltsville, MD. Consequently, correlations with flowering maturity did not include Beltsville, MD.

All metrics of PD were significantly related to one another (**Table 2**). Across environments, spiraling was highly correlated with visual dehiscence (ρ = 0.64 to 0.87) and moderately correlated to force to dehiscence (ρ = −0.17 to −0.42). Correlations between visual and force dehiscence were moderate (r = −0.14 to −0.43). Visual dehiscence exhibited a linear relationship with force to dehiscence, except for pods resisting dehiscence at high levels of force (> 4 N; **Figure 2**).

**Table 2:** Strength, significance, and consistency of association between pod dehiscence, morphology, and field data.

	Visual Dehiscence	Force to Dehiscence	Spiraling	Corrugation	Fracture	Flexibility	Pith Tissue	Flowering Maturity	Fall Vigor	Spring Vigor	Plant Weight	Seed Weight
Visual Dehiscence		Pearson	Polyserial/Kruskal-Wallis	Polyserial/Kruskal-Wallis	Polyserial/Kruskal-Wallis	Polyserial/Kruskal-Wallis	Polyserial/Kruskal-Wallis	Pearson	Pearson	Pearson	Pearson	Pearson
Force to Dehiscence	−0.33 ***		Polyserial/Kruskal-Wallis	Polyserial/Kruskal-Wallis	Polyserial/Kruskal-Wallis	Polyserial/Kruskal-Wallis	Polyserial/Kruskal-Wallis	Pearson	Pearson	Pearson	Pearson	Pearson
Spiraling	0.79 ***	−0.47 ***		Polychoric/χ^2^	Polychoric/χ^2^	Polychoric/χ^2^	Polychoric/χ^2^	Polyserial/Kruskal-Wallis	Polyserial/Kruskal-Wallis	Polyserial/Kruskal-Wallis	Polyserial/Kruskal-Wallis	Polyserial/Kruskal-Wallis
Corrugation	−0.56 ¥ ***	0.35 ¥ ***	−0.73 ¥ ***		Polychoric/χ^2^	Polychoric/χ^2^	Polychoric/χ^2^	Polyserial/Kruskal-Wallis	Polyserial/Kruskal-Wallis	Polyserial/Kruskal-Wallis	Polyserial/Kruskal-Wallis	Polyserial/Kruskal-Wallis
Fracture	0.39 ***	−0.041 NS	0.47 ***	−0.045 ¥ NS		Polychoric/χ^2^	Polychoric/χ^2^	Polyserial/Kruskal-Wallis	Polyserial/Kruskal-Wallis	Polyserial/Kruskal-Wallis	Polyserial/Kruskal-Wallis	Polyserial/Kruskal-Wallis
Flexibility	−0.44 ***	0.023 NS	−0.51 ***	0.26 ¥ ***	−0.61 ***		Polychoric/χ^2^	Polyserial/Kruskal-Wallis	Polyserial/Kruskal-Wallis	Polyserial/Kruskal-Wallis	Polyserial/Kruskal-Wallis	Polyserial/Kruskal-Wallis
Pith Tissue	−0.12 ¥ *	0.44 ***	−0.39 ¥ ***	0.35 ¥ ***	0.15 ¥ *	0.010 ¥ NS		Polyserial/Kruskal-Wallis	Polyserial/Kruskal-Wallis	Polyserial/Kruskal-Wallis	Polyserial/Kruskal-Wallis	Polyserial/Kruskal-Wallis
Flowering Maturity	−0.11 **	−0.09 *	−0.01 NS	0.06 † NS	0.073 NS	0.10 *	−0.10 †		Pearson	Pearson	Pearson	Pearson
Fall Vigor	−0.02	0.03	0.03	−0.02 ¥	0.00	−0.08	0.06 ¥	0.08		Pearson	Pearson	Pearson
Spring Vigor	−0.13 **	0.22 ***	−0.30 ***	0.18 ¥ **	−0.06	−0.05	0.40 ¥ ***	−0.14 **	0.28 ***		Pearson	Pearson
Plant Weight	−0.06	0.07 ¥	−0.14 ***	0.05 †	−0.10	−0.03	0.28 † ***	−0.44 ¥ ***	−0.12 **	0.28 ***		Pearson
Seed Weight	0.12 **	−0.37 ***	0.38 ***	−0.30 ¥ ***	0.13 *	−0.14 *	−0.37 ¥ ***	0.20 ***	0.13 **	−0.18 ***	0.29 ***	

*, **, and *** indicate significance of correlations at the 0.05, 0.01, and 0.001 probability level, respectively.

NS indicates that correlations are not significant at the α < 0.05 threshold.

† indicates only one observed paired environment comparison.

¥ indicates only three observed paired environment comparisons. Numbers show Pearson's product-moment correlation coefficient for continuous vs. continuous metrics, polyserial maximum likelihood estimates for dichotomous vs. ordinal or dichotomous metrics, and polychoric maximum likelihood estimates for dichotomous vs. dichotomous or ordinal metrics across environments. Significance values were calculated through Pearson's product-moment correlation for continuous vs. continuous metrics, chi-squared test of independence for dichotomous or ordinal vs. dichotomous or ordinal metrics, and Kruskal-Wallis one-way analysis of variance for continuous vs. dichotomous or ordinal metrics. Shading indicates consistency of the correlation among environments. Dark grey indicates significant differences in correlations among environments, with change in direction; light grey indicates significant differences in correlations among environments, but no change in direction; and white indicates no significant differences among environments).

PD was influenced by the environment where the pods developed. For the response of force to dehiscence, environment explained more variance (13.27%) than any pod morphology metrics (0% to 8.51%) or field maturity traits (0%, **Table 3**). Visual dehiscence was also influenced by environment (7.63% of variance), but to a lesser degree than force to dehiscence.

**Table 3:** Variance contribution of environment, pod morphology, and flowering maturity on metrics of dehiscence.

Visual Dehiscence			
Groups	Variance	Standard Deviation	% of Total Variance
Environment	0.068	0.26	7.63%
Corrugation	0.407	0.64	45.70%
Fracture	0.040	0.20	4.49%
Flexibility	0.021	0.15	2.41%
Pith Tissue	0.000	0.00	0.00%
Flowering Maturity	0.004	0.06	0.45%
Residual	0.350	0.59	39.32%
**Force to Dehiscence**			
Groups	Variance	Standard Deviation	% of Total Variance
Environment	0.120	0.35	13.27%
Corrugation	0.035	0.19	3.85%
Fracture	0.000	0.00	0.00%
Flexibility	0.000	0.00	0.00%
Pith Tissue	0.077	0.28	8.51%
Flowering Maturity	0.000	0.00	0.00%
Residual	0.673	0.82	74.37%

Visual dehiscence was most explained by corrugation, while force to dehiscence was explained by pith tissue, followed by corrugation.

Corrugation, pith tissue, fracturing structure, and flexibility of the pod tended to be related to metrics of PD. However, low frequency of corrugation and pith tissue at the northern environments limited the inference of these morphological correlations to only three southern environments. Pod corrugation had the highest correlation with PD among the studied morphology metrics. Higher levels of corrugation were associated with lower visual dehiscence (ρ = −0.33 to −0.54), and spiraling (ρ = −0.36 to −0.76) at all environments. Corrugation also accounted for 46% of variance in the random effects model with the response of visual dehiscence (**Table 3**), and 3.9% of variance with the response of force to dehiscence.

The presence of pith tissue in the pod was associated with more force required to split the pod (ρ = 0.12 to 0.48) and reduced spiraling (ρ = −0.16 to −0.27) among environments. In the random effects model with the response of force to dehiscence, pith tissue accounted for the most variance among pod morphology metrics. However, the variance explained by pith tissue was small (8.5%, **Table 3**).

The fracturing structure of the pod wall was related to spiraling at all environments (ρ = 0.23 to 0.52), and visual dehiscence at five of six environments (ρ = 0.23 to 0.46). Pod flexibility was moderately related to spiraling (ρ = −0.14 to −0.58), and to visual dehiscence at five of six environments (ρ = −0.27 to −0.63). In the random effects model with the response of visual dehiscence, fracturing structure and flexibility accounted for a small portion of variance, at 5.5% and 2.2%, respectively (**Table 3**).

Some measures of field performance were associated with PD. Spring vigor showed a weak correlation with visual dehiscence (r = −0.01 to −0.28), spiraling (ρ = −0.04 to −0.27), and force to dehiscence (r = −0.08 to 0.23), but the relationship varied by environment. Seed yield was correlated with spiraling at most sites (r = 0.08 to 0.56), force to dehiscence (r = 0.01 to −0.24) at some sites, and visual dehiscence at the southern sites (r = 0.13 to 0.47).

## Discussion

Our results indicated the potential to select for indehiscence in hairy vetch. Wide variation in visual and force to dehiscence existed among diverse genotypes. More importantly for selection, multiple lines exhibited indehiscence or very low levels of dehiscence.

This dataset also demonstrated environmental influence on PD, which is well documented in other species (see reviews by Ogutcen et al., 2018 and Zhang et al., 2018). Growing environment contributed substantial amounts of variance for visual dehiscence and force to dehiscence (**Table 3**). Moreover, correlations between metrics of PD, pod morphology, and flowering maturity significantly differed among environments (**Table 2**). To separate genetic effects from environmental influences and interactions, PD studies should utilize multiple environments. Secondary selection traits to speed phenotyping would need to consistently correlate among diverse environments within a breeding program region of interest.

Spiraling was highly correlated with PD and was a high-throughput measurement, requiring only 15 seconds per sample. Visual dehiscence provided higher resolution in PD than spiraling and was moderately time intensive, requiring 5 min to rate per line, at 50 pods evaluated per line. Force to dehiscence was the most involved measurement, requiring specialty equipment, a trained operator, and 18.5 min of evaluation time per line, with five pods evaluated per line. Although visual dehiscence and spiraling may be adequate to identify strongly dehiscent lines, force to dehiscence may be useful for identifying extreme lines most resistant to dehiscence. For initial screenings of dehiscence, spiraling could identify the genotypes most susceptible to PD at low cost. Visual dehiscence would be useful in early and middle stages of selection to eliminate moderately dehiscent lines. Once mean visual dehiscence levels become low (< 1) in a breeding population, force to dehiscence measurements would likely be necessary to further advance gains in selection (**Figure 2**).

Although pod morphology metrics were fast to measure (15 seconds per line), none were strongly related to PD among environments. Pod corrugation was moderately correlated with all measures of PD, and explained a large portion of variance for visual dehiscence. Pith tissue was moderately correlated with force to dehiscence and pod spiraling. However, pod corrugation and pith tissue did not commonly appear at the three environments in the northern United States. Consequently, pod corrugation and pith tissue would not be useful PD secondary selection traits for breeding programs including cold temperate climates.

Further study is needed to understand the physiology of pith tissue in hairy vetch pods. The pith tissue created a foam-like structure that seemed to inhibit compression force from breaking a pod, hence the trait's contribution to force to dehiscence (**Table 3**). However, the pith tissue may not be genetic resistance to PD, but rather a plant response to an environmental threat (e.g. a pathogen). To separate out environmental effects from true PD, the trait of pith tissue could serve as a covariate when analyzing force to dehiscence.

The fracture structure of the pod wall was moderately related to spiraling, and with visual dehiscence at some environments. The linear fracture morphology described in our paper likely relates to the alignment of pod wall fibers at an angle to pod sutures, which can cause spiraling of the carpel (Funatsuki et al., 2014). As the evaluation of spiraling required equal time to measure as fracture, and spiraling was more correlated to other metrics of PD, we see little utility for a rating of fracture.

Some traits showed inconsistent correlation with PD metrics among environments, such as pod flexibility. Such traits would not be reliable secondary selection traits for PD.

Pod moisture was not correlated with visual dehiscence or force to dehiscence. Consequently, pods in our study had likely reached the critical pod moisture required for PD. The weak correlation between spiraling and pod moisture could indicate that some samples were above the critical pod moisture threshold for PD. Moisture contents in our evaluation (6.7% to 9.3%) were below the critical pod moisture (10.1% to 10.4%) associated with PD in soybean (Zhang et al., 2018). However, these moisture contents were above the stable moisture found in common vetch (5%) (Dong et al., 2016). **Supplementary Figure 2** shows the results of PD after various pod drying times, heat conditions, and pod moistures to identify critical pod moisture in hairy vetch.

Flowering timing of lines were not strongly correlated with any measures of PD. In other species, genotypes with earlier flowering timing have exhibited more PD, as they were exposed for more time to heat and drying forces that can cause rupture of the dehiscence zone (Zhang et al., 2018). In our dataset, the stabilization of pod moisture *via* drying may have reduced the influence of maturity timing on PD.

Selecting for pod indehiscence may conflict with other field traits of interest in *Vicia villosa*. Lines with high spring vigor, a trait desired by growers (Wayman et al., 2016), also tended to have low PD, indicating the potential to select for both desired traits. However, there was a tradeoff between PD and seed yield in some environments. Selection for PD should closely monitor seed yield, to ensure lines developed for low PD also produce adequate yield for seed growers.

## Author's Note

Mention of trade names or commercial products in this publication is solely for the purpose of providing specific information and does not imply recommendation or endorsement by the U.S. Department of Agriculture.

## Data Availability Statement

The raw data supporting the conclusions of this article will be made available by the authors, without undue reservation, to any qualified researcher.

## Author Contributions

LK and HR designed and analysed the experiment. LK wrote results. BR, AB, NE, SK, SM, CR-H, MR, SE, SW, and NW acquired and assisted with interpretation of the data and critical revision for intellectual content. All authors provided approval of publication and agree to be accountable for all aspects of the work.

## Funding

Funding provided by National Institute for Food and Agriculture, Organic Research Extension Initiative Grant number 2015-51300-24192 and National Institute for Food and Agriculture Grant number 2018-67013-27570.

## Conflict of Interest

The authors declare that the research was conducted in the absence of any commercial or financial relationships that could be construed as a potential conflict of interest.
