# Supplementary material for: Pod Dehiscence in Hairy Vetch (Vicia villosa Roth)
Source: Front Plant Sci. 2020 Mar 3;11:82. doi: 10.3389/fpls.2020.00082 (PMC7063115; doi:10.3389/fpls.2020.00082)
Supplement: Supplementary file 1 [file DataSheet_1.docx]

Supplementary Material

# Supplementary Tables and Figures

## Supplementary Tables

**Supplementary Table 1.** Origin of hairy vetch breeding lines included in the pod dehiscence evaluation.

| **Maternal or grandmaternal line origin** | **Number of full-, half-, or quarter- sibling progeny evaluated** | **Reference or source information** |
| --- | --- | --- |
| Albert Lea Seed 2016 organic VNS | 35 |  |
| AU Early Cover | 5 |  |
| Groff | 8 |  |
| Hungvillosa | 10 |  |
| Buckwheat Growers 2016 VNS | 8 |  |
| MSP4045 | 23 | University of Minnesota breeding population |
| MSP4046 | 28 | University of Minnesota breeding population |
| MSP4047 | 14 | University of Minnesota breeding population |
| MSP4048 | 21 | University of Minnesota breeding population |
| MSP4049 | 43 | University of Minnesota breeding population |
| Landrace from David Podoll | 26 |  |
| Nebraska | 6 |  |
| Purple Bounty | 16 | US Government, 2013 |
| Purple Prosperity | 7 | US Government, 2013 |
| PI206492 | 8 | Godfrey, 1953 |
| PI220880 | 4 | Belgium Ministere de l'Agriculture, 1954 |
| PI263190 | 40 | N.I. Vavilov Research Institute of Plant Industry, 1960 |
| PI268321 | 20 | Harlan, 1960 |
| PI284100 | 5 | CSIRO, 1962 |
| PI284102 | 3 | Commonwealth Scientific and Industrial Research Organization, 1962 |
| PI284105 | 6 | Commonwealth Scientific and Industrial Research Organization, 1962 |
| PI491408 | 54 | Zhang, 1984 |
| PI493308 | 5 | Crespo, 1984 |
| PI536642 | 36 | N.I. Vavilov Research Institute of Plant Industry, 1989 |
| PI560503 | 10 | Sperling, Gecit, and Eser, 1989 |
| Savane | 8 | French breeding population |
| VIC051 | 26 | Serbian breeding population |
| VIC410 | 31 | Serbian breeding population |
| WIHV1 | 24 | USDA-ARS breeding population |
| WIHV2 | 11 | USDA-ARS breeding population |
| WIHV3 | 26 | USDA-ARS breeding population |
| WIHV4 | 14 | USDA-ARS breeding population |
| WIHV6 | 7 | USDA-ARS breeding population |
| WIHV7 | 12 | USDA-ARS breeding population |
| WIHV8 | 13 | USDA-ARS breeding population |

**Supplementary Table 2.** Correlations between pod moisture and pod dehiscence, pod morphology metrics, and field maturity timing. Pod moisture was not significantly correlated with visual dehiscence, force to dehiscence, pod morphology metrics or maturity timing at the threshold of α<0.05. Correlations were computed using Pearson’s correlation coefficient with continuous metrics and polyserial maximum likelihood estimates with dichotomous or ordinal metrics. Relationships were tested for difference from zero using Pearson’s product-moment correlation (Pearson’s) for continuous metrics and one-way analysis of variance (ANOVA) for dichotomous or ordinal metrics.

| **Trait** | **Correlation with Moisture** | **Significance of Correlation** | **Number of pairs** | **Correlation Coefficient** | **Significance Test** |
| --- | --- | --- | --- | --- | --- |
| Visual Dehiscence | -0.26 | 0.09338 | 42 | Pearson's | Pearson's |
| Force to Dehiscence | 0.054 | 0.7334 | 40 | Pearson's | Pearson's |
| Spiraling | -0.25 | 0.02048 | 76 | Polyserial | ANOVA |
| Corrugation | 0.2 | 0.2255 | 76 | Polyserial | ANOVA |
| Fracture | -0.0031 | 0.9832 | 76 | Polyserial | ANOVA |
| Flexibility | 0.21 | 0.1682 | 76 | Polyserial | ANOVA |
| Pith Tissue | -0.022 | 0.892 | 76 | Polyserial | ANOVA |
| Flowering Maturity | 0.046 | 0.6905 | 76 | Pearson's | Pearson's |

## Supplementary Figures

**Supplementary Figure 1.** Histogram of pods collected per maternal line from each site. Environments are listed as 18CL (Clayton, NC), 18GB (Goldsboro, NC), 18MD (Beltsville, MD), 18MN (St. Paul, MN), 18NYE (Ithaca, NY), 18NYR (Varna, NY), 18WI (Prairie Du Sac, WI).

**Supplementary Figure 2.** A calibration experiment determined that at least 24h at 30ºC was necessary to stabilize pod shatter and reach critical moisture in *Vicia villosa*. The experiment included progeny of five maternal lines that exemplified extreme values for shattering in both 2017 and 2018. All progeny were grown and harvested at one site (Knox City, TX) in 2019. One to three progeny from two high-shatter lines, two to three progeny from two inconsistent shattering lines, and three progeny from one low-shatter line were evaluated. A set of 200 pods from each of the 12 progeny lines was divided among two temperature treatments (30ºC or 38ºC), and three drying times (0h, 24h, 48h), and evaluated for shatter and moisture. Prior to drying, each sample was rated for visual shatter on a scale from 0 to 3, using the methods defined in Table 1. Samples were then placed in ovens at 30ºC or 38ºC for 24h or 48h time treatments. After each treatment, pods were removed, weighed, and again rated for visual shatter. Each sample was then dried at 105 ºC for 24h and reweighed to determine moisture content. All drying treatments had significantly different pod shatter ratings than the control of room temperature at p<0.05. There were no significant differences in drying times and temperatures among 30ºC, 38ºC, 24h, nor 48h treatments. The 24h 38ºC treatment had a lower pod moisture than the 0h control, although this result was not consistent at the 48h 38ºC treatment.

# References

Commonwealth Scientific and Industrial Research Organization. (1962). PI 284100 *Vicia villosa* Roth. USDA, U.S. National Plant Germplasm System <https://npgsweb.ars-grin.gov/gringlobal/accessiondetail.aspx?id=1211735> (accessed 29 September 2019).

Commonwealth Scientific and Industrial Research Organization. (1962). PI 284105 *Vicia villosa* Roth. USDA, U.S. National Plant Germplasm System <https://npgsweb.ars-grin.gov/gringlobal/accessiondetail.aspx?id=1211742> (accessed 29 September 2019).

Crespo, D.G. (1984). PI 493308 *Vicia villosa* Roth. USDA, U.S. National Plant Germplasm System <https://npgsweb.ars-grin.gov/gringlobal/accessiondetail.aspx?id=1388244> (accessed 29 September 2019).

Godfrey, R. K. (1953). PI 206492 Amorciras *Vicia villosa* Roth. USDA, U.S. National Plant Germplasm System <https://npgsweb.ars-grin.gov/gringlobal/accessiondetail.aspx?id=1173017> (accessed 29 September 2019).

Harlan, J.R. (1960). PI 268321 *Vicia villosa* Roth. USDA, U.S. National Plant Germplasm System <https://npgsweb.ars-grin.gov/gringlobal/accessiondetail.aspx?id=1201822> (accessed 8 July 2019).

Belgium Ministere de l'Agriculture. (1954). PI 220880 Vicia villosa Roth. USDA, U.S. National Plant Germplasm System <https://npgsweb.ars-grin.gov/gringlobal/accessiondetail.aspx?id=1178966> (accessed 29 September 2019).

N.I. Vavilov Research Institute of Plant Industry. (1960). PI 263190 Stanislan *Vicia villosa* Roth. USDA, U.S. National Plant Germplasm System <https://npgsweb.ars-grin.gov/gringlobal/accessiondetail.aspx?id=1199575> (accessed 8 July 2019).

N.I. Vavilov Research Institute of Plant Industry. (1989). PI 536642 Voronezskaja *Vicia villosa.* USDA, U.S. National Plant Germplasm System <https://npgsweb.ars-grin.gov/gringlobal/accessiondetail.aspx?id=1431578> (accessed 8 July 2019).

Sperling, C.R., Gecit, H.H., and Eser, D. (1989). PI 560503 *Vicia villosa* Roth. USDA, U.S. National Plant Germplasm System. <https://npgsweb.ars-grin.gov/gringlobal/accessiondetail.aspx?id=1455475> (accessed 29 September 2019).

U.S. Government as represented by the Secretary of Agriculture. (2013a). Hairy Vetch ‘Purple Prosperity’ Plant Variety Protection 200800302. Date issued: 13 December.

U.S. Government as represented by the Secretary of Agriculture. (2013b). Hairy Vetch ‘Purple Bounty’ Plant Variety Protection 200700280. Date issued 6 February.

Zhang, Y-F. (1984). PI 491408 *Vicia villosa* Roth. USDA, U.S. National Plant Germplasm System. <https://npgsweb.ars-grin.gov/gringlobal/accessiondetail.aspx?id=1386344> (accessed 29 September 2019).
